# Niosomes-loaded selenium nanoparticles as a new approach for enhanced antibacterial, anti-biofilm, and anticancer activities

**DOI:** 10.1038/s41598-022-26400-x

**Published:** 2022-12-19

**Authors:** Abbas Haddadian, Farnoush Falahi Robattorki, Hedieh Dibah, Ali Soheili, Erfan Ghanbarzadeh, Nasrin Sartipnia, Shadi Hajrasouliha, Kamal Pasban, Romina Andalibi, Mojtaba Hedayati Ch, Arezou Azari, Arman Chitgarzadeh, Aliasghar Bagheri Kashtali, Fatemeh Mastali, Hassan Noorbazargan, Amir Mirzaie

**Affiliations:** 1 Department of Biology, East Tehran Branch, Islamic Azad University, Tehran, Iran; 2 Biomedical Engineering Group, Chemical Engineering Department, Engineering Faculty, Tarbiat Modares University, Tehran, Iran; 3 Department of Biology, Roudehen Branch, Islamic Azad University, Roudehen, Iran; 4 Faculty of Medicine, Kermanshah University of Medical Sciences, Kermanshah, Iran; 5 Department of Microbiology, Faculty of Medicine, Guilan University of Medical Sciences, Rasht, Iran; 6 Department of Biology, Islamshahr Branch, Islamic Azad University, Islamshahr, Iran; 7 Department of Biology, Zanjan Branch, Islamic Azad University, Zanjan, Iran; 8 Department of Biotechnology, School of Advanced Technologies in Medicine, Shahid Beheshti University of Medical Sciences, Tehran, Iran; 9 Department of Biology, Parand Branch, Islamic Azad University, Parand, Iran

**Keywords:** Antimicrobials, Chemical biology, Microbiology

## Abstract

Targeted drug delivery and increasing the biological activity of drugs is one of the recent challenges of pharmaceutical researchers. Niosomes are one of the new targeted drug delivery systems that enhances the biological properties of drugs. In this study, for the first time, the green synthesis of selenium nanoparticles (SeNPs), and its loading into niosome was carried out to increase the anti-bacterial and anti-cancer activity of SeNPs. Different formulations of noisome-loaded SeNPs were prepared, and the physical and chemical characteristics of the prepared niosomes were investigated. The antibacterial and anti-biofilm effects of synthesized niosomes loaded SeNPs and free SeNPs against standard pathogenic bacterial strains were studied, and also its anticancer activity was investigated against breast cancer cell lines. The expression level of apoptotic genes in breast cancer cell lines treated with niosome-loaded SeNPs and free SeNPs was measured. Also, to evaluate the biocompatibility of the synthesized niosomes, their cytotoxicity effects against the human foreskin fibroblasts normal cell line (HFF) were studied using the MTT (3-[4,5-dimethylthiazol-2-yl]-2,5 diphenyl tetrazolium bromide) assay. The results illustrated that the optimal formulation had an average size of 177.9 nm, a spherical shape, and an encapsulation efficiency of 37.58%. Also, the results revealed that the release rate of SeNPs from niosome-loaded SeNPs and free SeNPs was 61.26% and 100%, respectively, in 72 h. Also, our findings demonstrated that the niosome-loaded SeNPs have significant antibacterial, anti-biofilm, and anticancer effects compared to the free SeNPs. In addition, niosome-loaded SeNPs can upregulate the expression level of *Bax*, *cas3*, and *cas9* apoptosis genes while the expression of the *Bcl2* gene is down-regulated in all studied cell lines, significantly. Also, the results of the MTT test indicated that the free niosome has no significant cytotoxic effects against the HFF cell line which represents the biocompatibility of the synthesized niosomes. In general, based on the results of this study, it can be concluded that niosomes-loaded SeNPs have significant anti-microbial, anti-biofilm, and anti-cancer effects, which can be used as a suitable drug delivery system.

## Introduction

Niosomes or non-ionic surfactant vesicles are very small layered structures that are obtained from a mixture of non-ionic surfactant alkyl or di-alkyl poly-glycerol ether, cholesterol, and then hydration in an aqueous medium^1^. Niosomes have a unique structure in that they encapsulate hydrophobic substances in their lipid part and hydrophilic substances inside the hydrophilic core^2^. Niosomes can trap different types of drugs, proteins, and vaccines and in recent years, they are among the new pharmaceutical systems on which many studies have been conducted^3,4^. Niosomes, like other vesicular systems, have many advantages, such as prolonging the residence time of the drug in the circulatory system, targeted drug delivery to specific organs and tissues, controlled release of the drug, and being biodegradable and non-immunogenic^5^.

One of the important applications of niosome is the targeted delivery of drugs to cancer and microbial cells. One of the most common cancers in the world is breast cancer^6,7^. Studies show that 8000 people are diagnosed with breast cancer in Iran every year, and the prevalence of breast cancer among Iranian women is about 30–35 cases per 1,000,000 people^8^. Usually, the common treatment for this cancer is chemotherapy, radiation therapy, and surgery. The use of these treatment methods has many side effects that reduce the quality of life of breast cancer sufferers^9^. Recently, researchers are looking for alternative therapy methods to control bacterial infections and cancers, and one of these approaches is the use of metallic nanoparticles^10^.

Among metallic nanoparticles, selenium nanoparticles (SeNPs) have attracted the attention of researchers and have special properties such as stability in environmental conditions and synthesis at low temperatures^11^. In general, there are various methods for the synthesis of SeNPs, one of these methods is the green synthesis method that is, the synthesis of nanoparticles using plant extracts. Physical and chemical methods are less used by researchers due to their high cost, low production efficiency, and environmental pollution^12^. To overcome this problem, the use of a green synthesis method using plant extracts has attracted the attention of researchers^13^. In this method, plant extracts are used as a reducing agent for the synthesis of SeNPs due to the presence of secondary and active compounds, which has a high production efficiency, are economically affordable, and are eco-friendly^14^. However, green synthesis methods are preferred due to their environmentally friendly, clean, safe, cost-effective, easy, and effective sources for high productivity and purity^15^. High pressure or temperature is not required for the green synthesis of nanoparticles, the use of toxic and dangerous substances and the addition of external reducing, stabilizing, or masking agents are avoided.

Various studies have been conducted on the green synthesis of SeNPs. Hashem et al. synthesized SeNPs using prickly pear peel waste extract and investigated its structure. The results of this study showed that the SeNPs had a spherical shape and a size between 10 and 87.4 nm and these researchers pointed out that the green synthesis method is easy and environmentally friendly. Salem et al. also showed that the green synthesis of SeNPs by orange peel waste extract is economical and eco-friendly and has good biological effects. Also, studies have shown that SeNPs have significant antimicrobial effects and the mechanism of antimicrobial effects is caused by metabolic interference, intracellular reactive oxygen species (ROS) concentration modulation, and bacterial membrane interruption. Also, due to interactions between SeNPs and various protein molecular structures, they have high adsorption and antimicrobial properties^15^. Lashin et al. synthesized SeNPs using *Ziziphus spinachristi* extract and studied its characterization and antimicrobial effects. The results of this study showed that the synthesized SeNPs had an average size between 20 and 45 nm and had profound antimicrobial activity against pathogenic Gram-positive and Gram-negative bacteria and multicellular fungi^16^. In our study, the extract of *Trifolium cherleri* was used to synthesize SeNPs. *T. cherleri* is a plant from the Fabaceae family, which is one of the flowering plants and is known as cup clover. This plant is native to the Canary Islands, the Mediterranean, and the Middle East to the east of Iran, and it has been introduced to Australia as fodder^17^. Recent studies revealed that the extract of this plant has secondary compounds such as terpenes, terpenoids, and flavonoids, which have reducing effects and are important in the green synthesis of nanoparticles. After that, the synthesized SeNPs were loaded into niosome, and their antimicrobial, anti-biofilm, and anticancer effects were investigated. In this study, SeNPs were synthesized by the green synthesis method using *T. cherleri* extract, and their characterization including size, morphology, crystallinity, and percent of SeNPs was performed. In addition, SeNPs were loaded into niosomes for the first time and their biological effects including antibacterial, anti-biofilm, and anti-cancer activities were studied.

## Materials and methods

### Materials

Chloroform, ethanol, Span 80, DCP, DMSO, cholesterol, SDS, and Amicon (Ultra-15-Membrane, MWCO 30,000 Da) were supplied from Merck, Germany. Trypsin-EDTA, Trypan blue, Medium RPMI-1640, DMEM, PBS, FBS, MTT, and Penicillin/Streptomycin 100 X were purchased from Gibco, USA. Dialysis membrane (MWCO 12,000 Da). Congo red, crystal violet solution, and all the other chemicals were purchased from Sigma-Aldrich Chemicals (St. Louis, MO). All the media were purchased from HiMedia Laboratories, India; the control bacterial strain was purchased from American Type Culture Collection (ATCC). HFF cell lines were obtained from Pasteur Cell Bank, Iran. RNA extraction kit was purchased from Qiagen, United States. Revert First Strand cDNA Synthesis Kit (Fermentas, Lithuania) was applied to synthesize the cDNA. All other chemicals and analytical grade solvents were also supplied from Merck (Germany).

### Green synthesis of SeNPs

We claim that all methods were performed under the relevant guidelines/regulations/legislation. In this study, *Trifolium cherleri* aerial parts were obtained from the plant bank of Iran Biological Reserves Center with herbarium number 1368 and were approved by a botanist. First, the plant was dried in conditions away from light and shade and completely powdered by an electric mill. After that, 10 g of dry plant powder was added to 50 mL of water and aqueous extraction was done by maceration method finally, the prepared extract was filtered with filter paper (Whatman, Germany). After that, for the green synthesis of SeNPs, 200 mL of sodium selenite solution (1 mM) was prepared, and 8 mL of the aqueous extract of the *T. cherleri* aerial parts extract was added to the sodium selenite solution, the reaction was carried out at room temperature and the color changed was achieved^18^. Then, the produced SeNPs were separated and purified using distilled water and centrifugation. The dried SeNPs were stored at room temperature for further analysis.

### GC/MS analysis

The analysis of gas chromatography connected to a gas chromatography-mass spectrometry (GC–MS) of the *T. cherleri* extract was performed with an Agilent 6890 device (made in the USA). The column type was DB-5, the column length was 30 m and the inner diameter was 0.25 mm for tracking, an electron ionization system with an energy of 70 eV was used. The thermal programming of the column was carried out from 50 to 250 °C with a temperature increase of 6 °C per minute. The carrier gas was helium 99.99% and the amount of injection was 1 µL and the gas flow rate was set at 15 mL/min. The volume of 1 µL of the plant extract was injected into the GC/MS machine and then the results obtained from the machine were identified by referring to the natural culture of natural compounds. Analysis of GC–MS spectra was performed using information available in the National Institute of Standards and Technology (NIST) database. Unknown mass spectra were compared using standard spectra available in the NIST library. The name, molecular weight, and compound structure of isolated substances were confirmed^19^.

### Preparation of niosome-loaded SeNPs

To prepare the niosome-loaded SeNPs, different amounts of cholesterol, Span 60 and Tween 60 were dissolved in a mixture of chloroform and methanol solvents (ratio 2:1) (Table 1). Then, the resulting solution was thoroughly stirred until the components were completely dissolved. The resulting solution is poured into a special rotary flask and placed under a vacuum under the desired conditions (60 °C and 150 rpm) until the solvent evaporates completely. Then, in the hydration stage, 10 mL of phosphate buffer containing SeNPs (1 mg/mL) was added to the prepared lipid film at a high phase transition temperature (60 °C) and using a rotary at 120 rpm rotated for half an hour for proper hydration. After finishing the hydration process, to reduce the particle size, sonication was performed for 7 min^20^.

**Table 1:** Various formulations of niosome-loaded SeNPs and the molar ratio of surfactant-cholesterol.

Formulation	Type of surfactant	Span60:Tween60 (mol ratio)	Lipid (µmol)	SeNPs (mg/mL)	Sonication time (min)	Surfactant:cholesterol (molar ratio)	Size (nm)	PDI	EE (%)-260 nm
F1	Span60	100:0	300	1	5	1:1	186.4	0.173	32.92
F2	Span60	50:50	300	1	5	1:1	177.9	0.154	37.58
F3	Span60	0:100	300	1	5	1:1	207.6	0.129	15.64
F2-B	Span60	50:50	300	–	5	1:1	122.5	0.137	–

### Encapsulation efficiency

Encapsulation efficiency (EE%) refers to the drug encapsulated in the niosome structure compared to the primary drug used. For this purpose, niosome formulation was centrifuged at 4 °C and 14,000*g* speed for 45 min. Niosomes-loaded SeNPs are precipitated and free SeNPs remain in the supernatant. The absorbance of the supernatant sample was read by a spectrophotometer at a wavelength of 265 nm, and the amount of free SeNPs was calculated and subtracted from the initial amount of the initial SeNPs, and from that, the EE% was calculated:

$${\text{Encapsulation}}\,{\text{efficiency}} = {\text{amount}}\,{\text{of}}\,{\text{free}}\,{\text{drug}} - {\text{the}}\,{\text{amount}}\,{\text{of}}\,{\text{primary}}\,{\text{drug}}/{\text{amount}}\,{\text{of}}\,{\text{primary}}\,{\text{drug}} \times {1}00$$

Also, various niosome formulations were prepared based on the molar concentration of Span 60/Tween 60, and the size of the synthesized niosomes and the EE% were also determined^21^.

### Physical and chemical properties of synthesized niosomes

Scanning Electron Microscope (SEM) was used to examine the morphological characteristics of the synthesized niosome-loaded SeNPs. Also, to determine the size, dynamic light scattering (DLS) was used, and the infrared spectroscopy (FTIR) analysis method was used to examine the functional groups of the surface of the produced niosomes^22^. Also, UV–Vis at wavelengths of 200–700 nm, X-ray diffraction (XRD), and Transmission electron microscopy (TEM) were used for the characterization of SeNPs.

### Drug release test

Drug release is evaluated dynamically. In this way, 2 mL of niosome-loaded SeNPs and also free SeNPs are placed in the dialysis bag (molecular weight cutoff 12 KDa) separately. Each of the bags is suspended in a flask containing 50 mL of phosphate-buffered saline (PBS) at 37 °C. The flasks with the bag containing the niosome containing SeNPs and the free SeNPs are placed on the stirrer. Sampling is done at different hours, in such a way that 1 mL of PBS containing the dialysis bag is removed and 1 mL of PBS with a temperature of 37 °C is replaced. Sampling is done up to 72 h in specific time intervals (1, 2, 4, 8, 24, 48, and 72 h). The optical absorption of the samples was read by the UV spectrophotometer at a wavelength of 265 nm and the graph of the cumulative release percentage of SeNPs from niosome was drawn for 72 h^23^.

### Stability studies

To check the stability, the optimal sample was kept for 1 month in both room and refrigerator temperatures and was evaluated in terms of %EE and size at specific time intervals^24^.

### Antimicrobial activity

To investigate the antimicrobial effects of niosome-loaded SeNPs and free SeNPs, the lowest inhibitory concentration (MIC) and lowest bactericidal concentration (MBC) method was used. MIC and MBC tests based on CLSI (Clinical and Laboratory Standards Institute) guidelines by dilution method in triplicated for niosome loaded SeNPs and free SeNPs against pathogenic microbial strains *Staphylococcus aureus* ATCC 25923, *Enterococcus faecalis* ATCC 29212, *Pseudomonas aeruginosa* ATCC 39615 and *Escherichia coli* ATCC 25922. Add 5 µL of microbial culture with 0.5 McFarland concentration and 95 µL of Mueller Hinton Broth culture medium to all wells. After that, concentrations of 3.125 to 100 µg/mL of niosome-loaded SeNPs and free SeNPs were added to the wells. Finally, MIC and MBC were considered the lowest concentration of bacterial growth inhibitors and lowest lethal concentrations. In addition, Mueller Hinton Broth culture medium without bacteria was used as a negative control, and a well containing only standard bacteria was used as a positive control^25^.

### Time kill assay

The antibacterial activity of niosomes-loaded SeNPs, free SeNPs, and free niosome against determined pathogenic bacteria was studied within 72 h using the time-kill assay method in a 96-well plate. Briefly, 100 μL of niosome-loaded SeNPs and free SeNPs at their sub-MIC concentrations were added to plate wells pre-loaded with 100 μL of each 10^5^ CFU/mL bacterial suspension. After incubation at 37 °C, the optical absorbance of samples at OD 600 nm was measured at 2, 4, 6, 24, 48, and 72 h using a microplate reader^26^.

### Anti-biofilm effects

To evaluate the anti-biofilm effects of niosome-loaded SeNPs, free SeNPs, and free niosome, the microtitre plate method using crystal violet (CV) in 96-well plates was used. The standard strains were cultured in 96-well plates for 24 h at 37 °C. After that, all the wells were washed three times with PBS to wash the unattached strains from the bottom of the wells. Standard strains were then treated with a sub-MIC concentration of niosome-loaded SeNPs and free SeNPs for 24 h at 37 °C. Then all the wells were washed again with PBS three times and fixed with methanol for 15 min. The 96-well plates were air-dried for 30 min and 100 µL of 0.1% CV solution were added to each well and incubated for 20 min at room temperature. After washing with distilled water, 100 μL of 33% acetic acid was added to each well, and the optical absorbance was measured at 570 nm. The average absorption values of each sample were calculated and compared with the average values of the control. The control samples in this test were strains not treated with niosome-loaded SeNPs and free SeNPs^27^.

### Evaluation of biofilm gene expression level

The standard bacterial strains were treated with the sub-inhibitory concentration (Sub-MIC) of niosome-loaded SeNPs, free SeNPs, and free niosome, and then, RNA extraction from the treated strains was performed using the RNA extraction kit (Qiagen, USA) according to the instructions. cDNA synthesis was performed using the Quanti Tect Reverse Transcription kit (Fermentas, Lithuania). The quantitative Real-Time-PCR (qRT-PCR) method was used to investigate the expression of biofilm genes including *icaD*, adhesion of collagen of *E. faecalis* (*Ace*), *FimH*, and *pelF*. Also, the *16S rRNA* gene was used as a housekeeping gene (internal control). Finally, the relative expression of biofilm-related genes was calculated by the ΔΔCт method. The sequence of used primers is given in Table 2.

**Table 2:** The primer sequences of biofilm-related genes.

Gene	Primer sequence (5′–3′)	Ref
*icaD*	F ATGGTCAAGCCCAGACAGAG R AGTATTTTCAATGTTTAAAGCAA	^25^
*Ace*	F GGAGAGTCAAATCAAGTACGTTGGTT R TGTTGACCACTTCCTTGTCGAT	^26^
*FimH*	F CATGCCATGGCCATGAAACGAGTTATTACC R CCCAAGCTTTTGATAAACAAAAGTCAC	^27^
*pelF*	F GAGGTCAGCTACATCCGTCG R TCATGCAATCTCCGTGGCTT	^28^
*16S rRNA*	F TATGGAGGAACACCAGTGGCGAAG R TCATCGTTTACGGCGTGGACTACC	^29^

### Cell toxicity and biocompatibility test

Cytotoxicity of niosome-loaded SeNPs, free SeNPs, and free niosome was studied using a colorimetric assay (MTT) against three breast cancer cell lines including MCF-7, T47D, and MDAMB231. For this purpose, cell lines were obtained from the Pasteur Institute Iran cell bank of Iran (Tehran-Iran). Then, the cells were seeded separately at the density of 10^4^ cells per well in a 96-well plate for 24 h. Then, the cells were treated with different concentrations of niosome-loaded SeNPs and free SeNPs (3.125 to 100 μg/mL) and after 48 h of incubation, MTT dye solution (5 mg/mL in PBS) was added to the wells and kept in the incubator for 3 h. The supernatant solution was removed and 100 µL of dimethyl sulfoxide (DMSO) solution was added to it, and the absorbance of all the wells was read at 570 nm wavelength, and according to the following formula, the survival percentage of the cells was calculated:

$${\text{Cell}}\,{\text{survival}}\,\% = {\text{optical}}\,{\text{absorbance}}\,{\text{of}}\,{\text{treated}}\,{\text{cells}}/{\text{optical}}\,{\text{absorbance}}\,{\text{of}}\,{\text{control}} \times {1}00.$$

Also, to check biocompatibility, the cytotoxicity of empty niosomes against the HFF normal cell line was investigated by the MTT method^30^.

### The expression level of apoptotic genes

In this study, to investigate the expression of apoptotic genes including *Bax* and *Bcl2*, it was measured by the Real-Time PCR method. At first, the total RNA of treated and untreated cells with niosome-loaded SeNPs and free SeNPs was extracted using the RNA extraction kit (Qiagen, USA) according to its instructions. The synthesis of complementary DNA was done with a cDNA synthesis kit (Fermentas, Lithuania). To perform Real-Time PCR, the specific primers of target genes *Bax*, *Bcl2*, *cas3*, *cas9*, and *GAPDH* gene (Housekeeping gene) were used as an internal control. The reverse primer sequence of the *Bax* target gene was 5′-TTGCTTCAGGGTTTCATCCAG-3′, and reverse 5′-AGCTTCTTGGTGGACGCATC-3′. The reverse primer sequence of *Bcl2* target gene was 5′-TGTGGATGACTGAGTACCTGAACC-3′, and reverse 5′-CAGCCAGGAGAAATCAAACAGAG, and for GAPDH reference gene as 5′-CGTCTGCCCTATCAACTTTCG-3′, and reverse 5′-CGTTTCTCAGGCTCCCCTCT-3′ and *cas 3* 5′-CATACTCCACAGCACCTGGTTA-3, reverse R 5′-ACTCAAATTCTGTTGCCACCTT-3′, *cas 9* 5′-CATATGATCGAGGACATCCAG-3, reverse R 5′-TTAGTTCGCAGAAACGAAGC-3′.

### Statistical analysis

All the tests of this study were repeated 3 times and the results were analyzed by GraphPad Prism (version 8) software using a one-way analysis of variance and *p* < 0.05 was considered significant.

### Ethics approval

All protocols were performed in accordance with the Ethical Committee and Research Deputy of the Islamic Azad University, Parand, Iran (IR.IAU.PAR.REC.1401.029).

## Results and discussion

### Extraction, GC–MS analysis, and green synthesis of SeNPs

**Figure 1:**
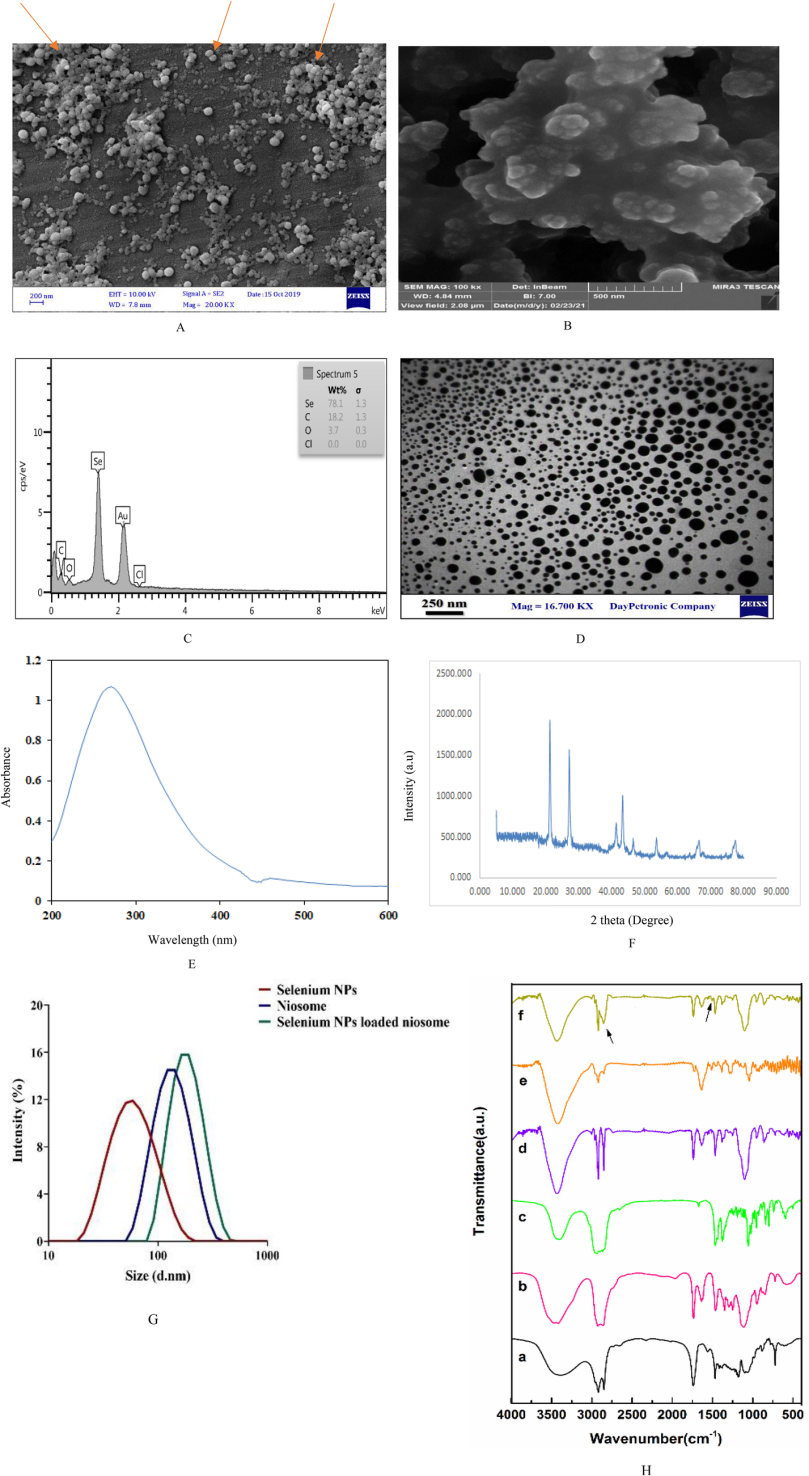
The synthesized SeNPs and niosome-loaded SeNPs characteristics. (**A**) SEMof green synthesized SeNPs, (**B**) SEM of noisome loaded SeNPs, (**C**) EDX analysis of synthesized SeNPs, (**D**) TEM of SeNPs, (**E**) UV–vis spectra of biosynthesized SeNPs, (**F**) XRD analysis of SeNPs, (**G**) DLS analysis of niosome loaded SeNPs, (**H**) FT-IR spectra of Tween 60 (a), Span 60 (b), Cholesterol (c), Niosome (d) and SeNPs (e) and Niosome-loaded SeNPs (f).

The dried powder of the *T. cherleri* was mixed in solvent for 24 h and after extraction, the resulting extract was filtered through Whatman paper. The reduction reaction of the sodium selenite solution took place at room temperature after adding the *T. cherleri* extract to the sodium selenite solution and a color change was obtained (from yellow to reddish), which indicated the formation of SeNPs. To confirm the synthesis of SeNPs, scanning electron microscopy and Energy Dispersive X-Ray Analysis (EDX) were used for the elemental chemical composition of SeNPs. The results showed that the synthesized SeNPs are spherical (Fig. 1A) and the amount of elemental selenium is 78.1% (Fig. 1B). UV–Vis spectroscopy at different wavelengths Between 200 and 700 nm was used to investigate the biosynthesis of Se-NPs. As shown in Fig. 1E, the maximum absorption peak is observed at the wavelength of 275 nm, which indicates the biosynthesis of selenium nanoparticles. Morphological characteristics of biosynthesized SeNPs were investigated using TEM. The results showed that the synthesized SeNPs have a spherical shape (Fig. 1D). The XRD pattern of SeNPs is represented in Fig. 1F, which demonstrated the hexagonal structure of the formed SeNPs. The typical diffraction peaks lied at 2 h = 66.21, 55.49, 50.07, 43.35, 40.41, 29.61, and 23.17, were ascribed to (210), (202), (112), (102), (110), (101) and (100) planes of the SeNPs crystalline form.

GC–MS chromatogram analysis of the *T. cherleri* extract showed 20 peaks, which indicated the presence of phytochemical compounds in the extract. By comparing the spectra with NIST library data, 20 chemical compounds were identified (Table 3). Among the identified compounds, Hexadecanoic acid, ethyl ester (20.7%), and 2-pentadecanone, 6,10,14-trimethyl (19.9%) had the highest percentage of ingredients. GC–MS analysis shows that the aerial part extract of *T. cherleri* has a source of flavonoids and phenolics that have reducing functional groups. These functional groups may play a role in the reduction of sodium selenite solution to SeNPs. The biomolecules in *T. cherleri* extract can act as an active biocatalyst to stabilize the produced SeNPs. Green synthesis using plant extract reduces toxic chemicals and leads to the synthesis of environmentally friendly nanoparticles^31^. Hashem et al. synthesized SeNPs using *Bacillus megaterium* and investigated its physico-chemical properties. The results of size measurement using the DLS method showed that the SeNPs had an average size of 41.2 nm with a spherical shape^32^.

**Table 3:** GC–MS analysis of *T. cherleri* extract.

No	Compound identified	Molecular formula	MW	RT (min)	Peak area (%)	Structure
1	Undecane, 4,6-dimethyl	C_13_H_28_	184	5.708	1.100	
2	Nonane, 2,2,4,4,6,8,8-heptamethyl	C_16_H_34_	226	6.057	3.129	
3	2(4H)-Benzofuranone, 5,6,7,7a-tetrahydro-4,4,7a-trimethyl	C_11_H_16_O_2_	180	7.756	1.070	
4	Heneicosane	C_21_H_44_	296	8.848	1.252	
5	Octadecane	C_18_H_38_	254	9.406	2.111	
6	3,7,11,15-Tetramethyl-2-hexadecen-1-ol	C_20_H_40_O	296	9.804	11.906	
7	2-Pentadecanone, 6,10,14-trimethyl	C_18_H_36_O	268	9.993	19.902	
8	3,7,11,15-Tetramethyl-2-hexadecen-1-ol	C_20_H_40_O	296	10.237	5.003	
9	Tetradecane, 2,6,10-trimethyl	C_17_H_36_	240	10.573	3.207	
10	8-Hexadecenal, 14-methyl-, (Z)	C_17_H_32_O	252	10.775	10.608	
11	Sulfurous acid, butyl heptadecyl ester	C_21_H_44_O_3_S	376	10.994	1.605	
12	Hexadecanoic acid, ethyl ester	C_18_H_36_O_2_	284	11.392	20.727	
13	Linoleic acid ethyl ester	C_20_H_36_O_2_	308	12.999	2.150	
14	1,5,9,13-Tetradecatetraene	C_14_H_22_	190	13.107	1.788	
15	Hexadecanoic acid, ethyl ester	C_18_H_36_O_2_	284	13.218	1.502	
16	7,9-Dihydroxy-6,9a-dimethyl-3-methylenedecahydroazuleno[4,5-b]furan-2(3H)-one	C_15_H_22_O_4_	266	14.229	2.312	
17	Pentasiloxane, dodecamethyl	C_12_H_36_O_4_Si_5_	384	14.389	1.130	
18	Hexasiloxane, tetradecamethyl	C_14_H_42_O_5_Si_6_	458	14.897	2.522	
19	Heptasiloxane, hexadecamethyl	C_16_H_48_O_6_Si_7_	532	16.130	2.680	
20	Phthalic acid, 6-ethyloct-3-yl 2-ethylhexyl ester	C_26_H_42_O_4_	418	16.466	2.675	

### Synthesis of niosome-loaded SeNPs and their physical and chemical characteristics

In this study, the optimal formulation of niosome-loaded SeNPs was synthesized with a molar ratio of 2:1 (surfactant/cholesterol) and a molar ratio of 50:50 (Span 60/Tween 60) with a concentration of 1 mg/mL of the SeNPs (Tables 1). Based on the results of Table 1, the F2 formulation was optimal so that it had a higher EE% and smaller size compared to other formulations. Therefore, this formulation was used to continue the study. Studies have shown that the effect of cholesterol concentration on the amount of drug encapsulation depends on various factors^33^. By increasing the amount of cholesterol, the lipophilicity and stability of the two layers’ increases, and the permeability decreases, so the drug can be more effectively trapped in the two layers of the vesicle^34^. But the large increase in the amount of cholesterol causes competition between the drug and cholesterol to be placed in the space between the two layers of niosomes, and the drug remains in the structure without encapsulation^35^. Cholesterol is one of the common materials used for the synthesis of stable niosomal formulations^36^. Studies show that cholesterol stabilizes bilayers, prevents leakage, and delays the penetration of solutes enclosed in the aqueous core of these vesicles^37^. Further, SEM and DLS were used to investigate the morphological characteristics of the synthesized niosomes. The results of the SEM show that the synthesized niosomes have a spherical structure (Fig. 1B). Also, the DLS results showed that the optimal size of the synthesized niosomes is 177.9 nm (Table 1, Fig. 1G). The FTIR results indicate the peaks related to the functional groups belonging to the niosome compounds, including the 1096 cm^−1^ peak related to the stretching C–O alcohol bond in the structure of cholesterol and Span 60. Also, the peaks of 1044 cm^−1^ and 1278 cm^−1^ correspond to SeNPs (Fig. 1, Table 4). In some studies, the synthesis of SeNPs, and the loading of nanoparticles in niosomes have been carried out, and their results are consistent with the results of our study. Swati De et al. synthesized niosomes loaded gold nanoparticles and investigated their properties using electron microscopy and DLS. Their results showed that the synthesized niosomes are spherical and the size of the synthesized niosomes is between 100 and 2000 nm^37,38^. Cittrarasu et al. prepared SeNPs using the green synthesis method and studied their size and morphology characteristics using Field Emission Scanning Electron Microscope (FE-SEM). The results showed that the synthesized SeNPs have a uniform spherical morphology and an average size of 55.9 nm^39^. One of the reasons for the different sizes of synthesized SeNPs can be the reducing properties of plant extracts used in green synthesis methods. Some plant extracts have more secondary compounds such as terpenes, flavonoids, tannins, coumarins, cinnamic acid, phenolic acid, vitamins, sterols, polysaccharides, enzymes, proteins, etc., and have more reducing capacity.

**Table 4:** The main characteristic peaks for FT-IR spectra of different samples/chemicals.

Sample, chemicals	Peak cm^−1^	Description
Tween 60	1117	C–O stretching
	1730	C=O stretching
	2860–3907	C–H stretching
	3452	OH stretching
Span 60	1162	C–O stretching
	2852–2917	C–H stretching
	3400	OH stretching
Cholesterol	1717	C=O stretching
	2800–2890	C–H stretching
	3398	OH stretching
	1025–1364	CH_2_ bending and CH_2_ deformation
	1455	C–C stretching in aromatic ring
	1664	C=C stretching
Niosome	1110	C–O stretching
	1736	C=O stretching
	2858–2923	C–H stretching
	3406	OH stretching
	1000 to 1292	Aliphatic C–N stretching
Selenium	1044	C–O stretch
	1278	=O stretch bend
	1513	=CH asymmetric

### Drug release pattern

**Figure 2:**
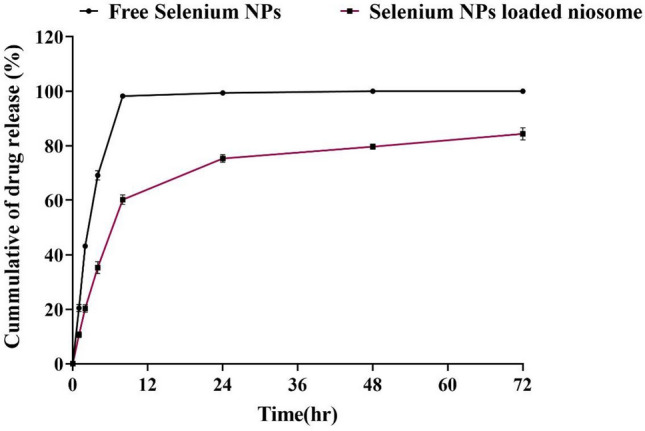
The release profile of SeNPs from noisome-loaded SeNPs at 72 h. SeNPs release from niosomal form has been compared with free SeNPs.

Figure 2 shows the cumulative release process of the free form of the SeNPs and the niosome-loaded SeNPs in the release medium of PBS for 72 h. To simulate and bring the ex vivo release environment closer to the real and in vivo conditions, the PBS release medium was used for the receptor phase, the release of the SeNPs from the niosomal form (61%) is less than the free SeNPs (100%) during the release period (72 h). In the release of free SeNPs, 92% of the SeNPs were released in the medium during the first 8 h, but for the noisome-loaded SeNPs, 37% of the SeNPs were released from the niosomal form within 8 h of release. According to the results, drug release from niosome occurs in 2 steps. The initial stage of 0–8 h, when the release of SeNPs is very fast and explosive, diffuses SeNPs to the release medium. The second stage is the slow-release stage, in which SeNPs slowly diffuse to the release medium for 72 h. In general, studies show that drug release from niosomes is explosive in the first hours (first 8 h) and gradually decreases in the following hours. Also, the release speed depends on the composition of the niosome, niosomes with stiffer vesicles that produce more slowly. The main composition of the F2 formulation was Span 60, Tween 60 with cholesterol, which is expected to produce a rigid vesicle membrane. Also, several studies showed that the use of Tween 80 in the preparation of niosomes causes their fluidity and the release rate of the vesicular membrane increases^40^. The release characteristics of SeNPs from niosomes potentially meet the requirements of an in-use antimicrobial delivery system. A relatively rapid release of SeNPs in the first 8 h is required at effective concentrations to inhibit initial colonization by bacteria, while a slower release allows the antimicrobial effect to be maintained for longer hours and inhibits microbial growth.

### Stability study

**Figure 3:**
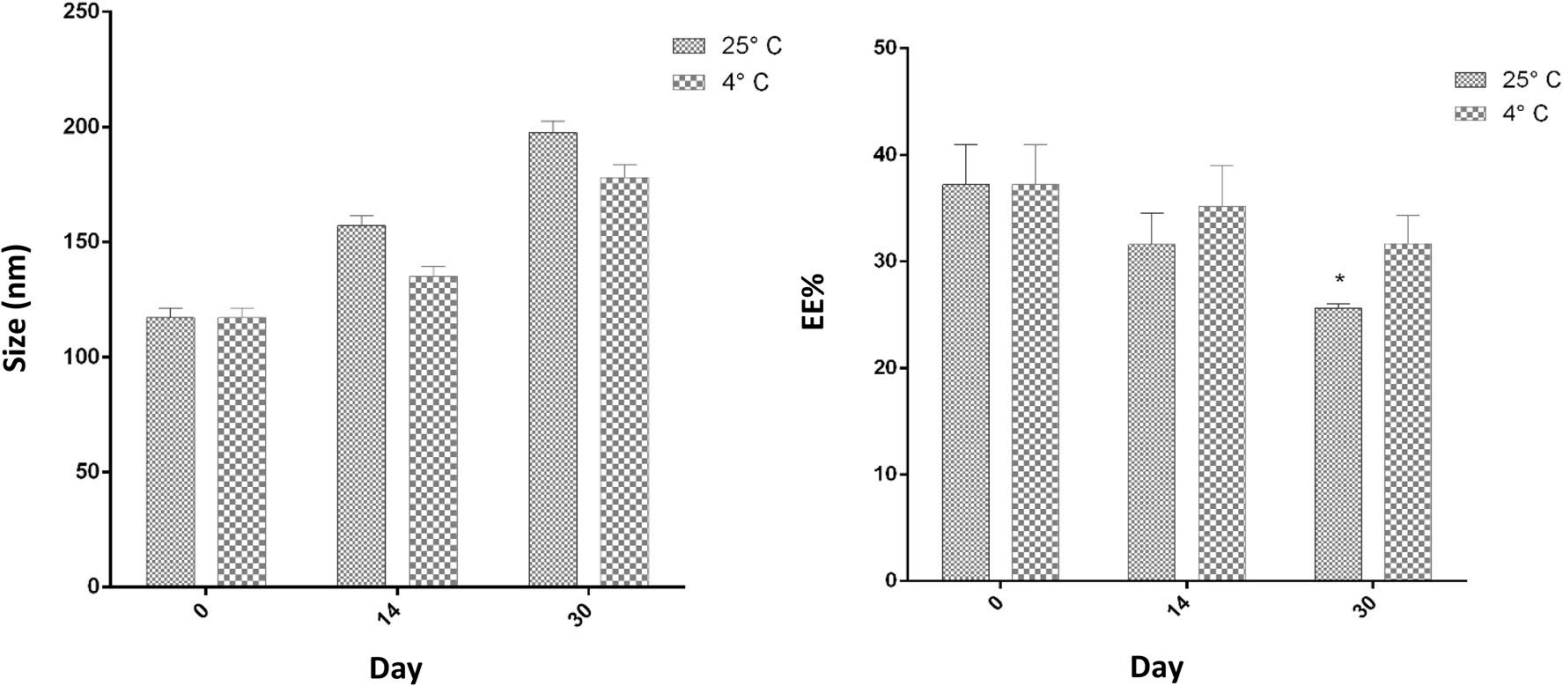
Stability of niosome-loaded SeNPs at two temperatures of 4 °C and 25 °C based on size (nm) and EE%. n = 3.

To check the stability of the optimal sample, three samples were kept at 4 °C and three samples at 25 °C and the size and EE% of the samples were checked at specific time intervals and the results were reported as their average. As shown in Fig. 3, the samples stored in the refrigerator compared to the samples stored outside have better stability in terms of size and EE% and had the least change in value during 30 days in terms of the mentioned conditions. This means that the increase in size and decrease in the encapsulation efficiency of the samples kept in the refrigerator were slower than the samples kept in the environment. Also, the stability results in two storage conditions show that with time, the amount of size increases, and the amount of encapsulation efficiency decreases. As can be seen, there is a significant difference between the sizes of the samples kept at 14 and 30 days between the two investigated temperatures. Also, there is a significant difference between the samples kept in the two studied conditions on the 30th day in terms of the EE% (Fig. 3).

### Antimicrobial activity

Antibacterial effects of free SeNPs, niosome-loaded SeNPs and free niosome with concentrations of 3.125–100 µg/mL on *Staphylococcus aureus*, *Enterococcus fecalis*, *Escherichia coli*, and *Pseudomonas aeruginosa* with microdilution method were performed. The results indicated that the niosome-loaded SeNPs have significant antibacterial properties on all the studied bacteria so that the MIC and MBC values of the niosome-loaded SeNPs were related to *Staphylococcus aureus* and the highest was related to *Pseudomonas aeruginosa*. As the results show, niosome-loaded SeNPs had more significant antimicrobial effects than free SeNPs, so the MIC and MBC values decreased between 2 and 4 times (Table 5). Also, the results showed that free niosome had no antimicrobial effects. It seems that the direct contact and fusion of the niosome with the bacterial membrane can increase the antimicrobial activity of the niosome-loaded SeNPs^41,42^. Various studies have been conducted on investigating the antimicrobial effects of SeNPs and niosomes containing antimicrobial compounds.

**Table 5:** Antibacterial activity of free SeNPs and niosome loaded SeNPs against selected pathogenic bacteria.

Bacteria	MIC/MBC of free SeNPs (µg/ml)	MIC/MBC of niosome-loaded SeNPs (µg/ml)	SubMIC value of free SeNPs/noisome-loaded SeNPs (µg/ml)
*Staphylococcus aureus*	12.5/25	3.125/6.25	6.25/ > 3.125
*Enterococcus fecalis*	25/50	3.125/6.25	12.5/ > 3.125
*Escherichia coli*	50/100	12.5/12.5	25/6.25
*Pseudomonas aeruginosa*	50/50	3.125/6.25	25/ > 3.125

Mansouri et al. studied the antimicrobial effects of niosomes loaded streptomycin against some pathogenic strains. The results of this study showed that niosomes containing streptomycin can reduce MIC value by 4–8 times, which indicated the significant effects of niosomes containing streptomycin^43^. Hedayati et al. encapsulated tobramycin in niosomes and studied its antimicrobial effects against *Pseudomonas aeruginosa* strains. The results of this study revealed that niosome containing tobramycin had significant antimicrobial effects compared to free tobramycin and reduced antibiotic resistance^44^. Targhi et al. synthesized niosomes loaded Curcumin-Cu and curcumin-Ag and investigated their antimicrobial effects against *S. aureus* and *P. aeruginosa* strains. The results of this study showed that niosomes can significantly reduce MIC and MBC values and had significant antimicrobial effects against selected pathogenic bacteria^45^. The results of these studies are in line with our findings and the researchers stated that niosomes can increase antimicrobial effects and be used as a candidate drug delivery system.

### Time kill assay

**Figure 4:**
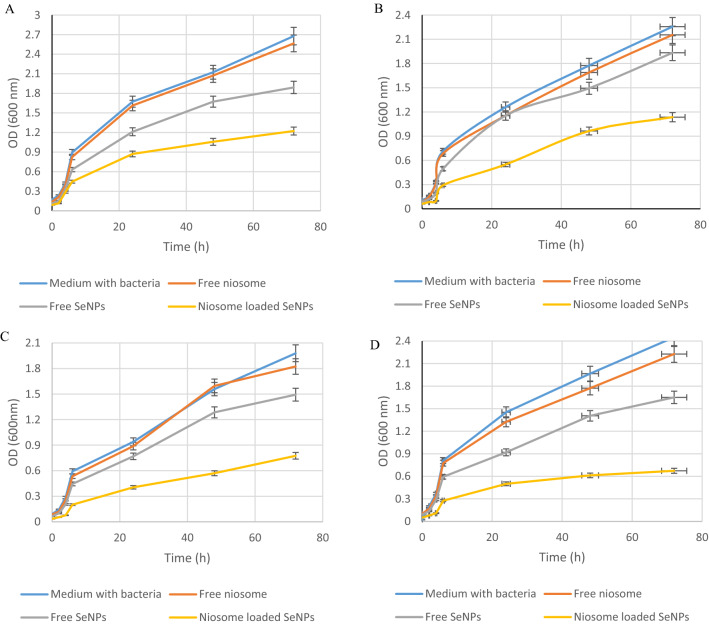
Antibacterial activity of free SeNPs and niosome loaded SeNPs against selected pathogenic bacteria including *P. aeruginosa* (**A**), *E. coli* (**B**), *E. fecalis* (**C**), and *S. aureus* (**D**).

To study the killing profile of niosomes-loaded SeNPs and free SeNPs, we used the time-kill assay method against *S. aureus*, *E. fecalis*, *E. coli*, and *P. aeruginosa* strains. In this test, we used the sub-MIC concentration of niosome-loaded SeNPs and free SeNPs for 72 h. In 72 h, as can be seen, the antibacterial effects of niosome loaded SeNPs are significantly higher than those of free SeNPs (Fig. 4). Heidari et al. studied the antimicrobial effects of niosomes containing tannic acid against *Escherichia coli*, *Klebsiella pneumoniae*, *Pseudomonas aeruginosa*, and *Staphylococcus aureus* strains using a time-kill assay. The results of this study showed that niosomes containing tannic acid can inhibit microbial growth in a slow and controlled manner in 72 h, but the free drug was used in the early hours and its concentration decreased^25^. Sadeghi et al. synthesized the niosome containing Lysostaphin-LL-37 and studied its antimicrobial effects against *S. aureus* strains. The results of this study indicated a slow and long-lasting inhibition for niosome-loaded lysostaphin-LL-37 within 72 h, while the free drug was used in the early hours and its concentration was exhausted^46^.

### Anti-biofilm activity

**Figure 5:**
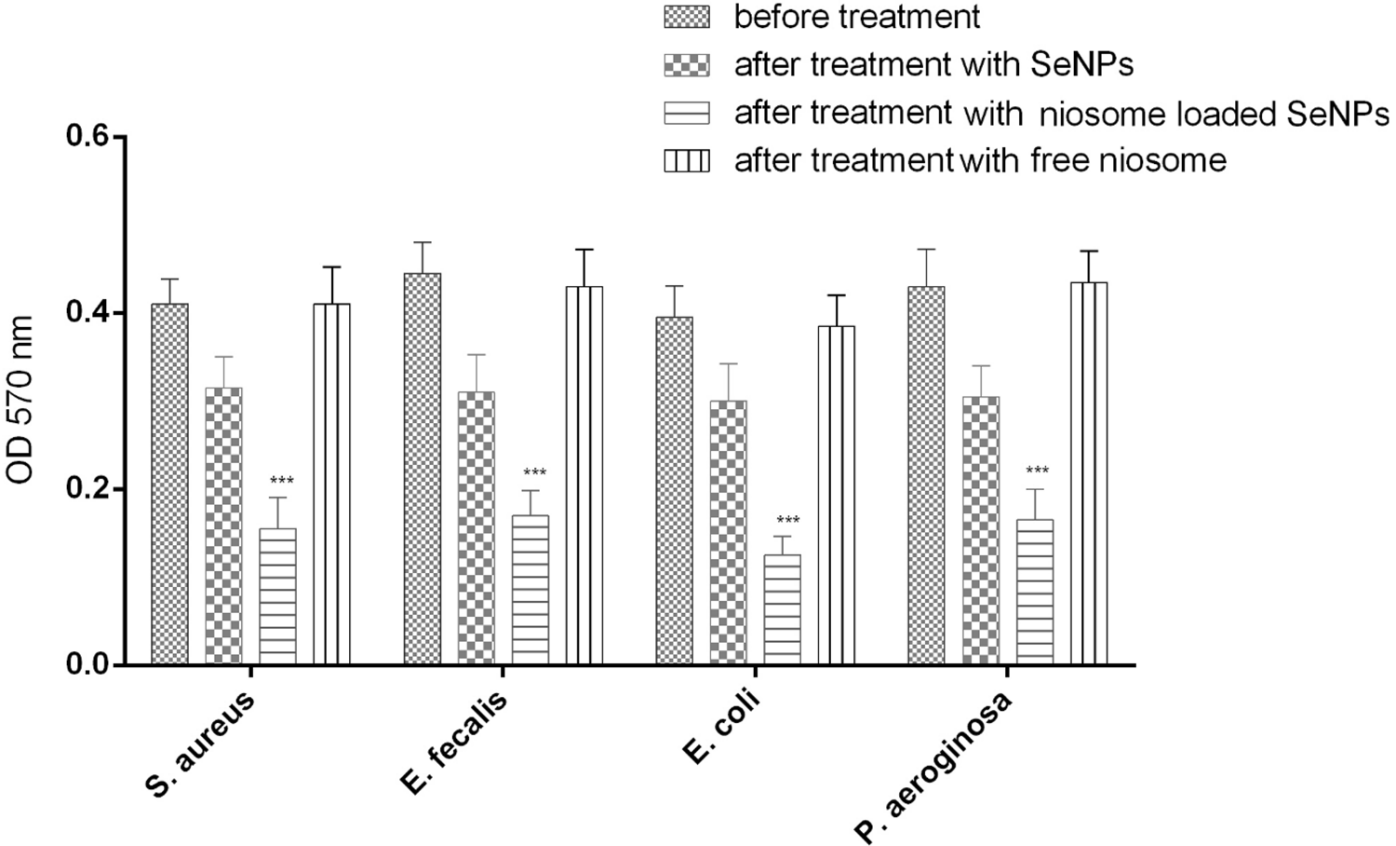
Anti-biofilm activity of free SeNPs, free niosome, and noisome-loaded SeNPs. Data represent the mean ± SD (n = 3). Error bars represent standard deviations. ***p < 0.001.

To investigate the anti-biofilm effects of niosome-loaded SeNPs, free niosome, and free SeNPs, the crystal violet method was used. As seen in Fig. 5, niosomes-loaded SeNPs have more significant anti-biofilm effects than free SeNPs. Also, the results showed that niosomes-loaded SeNPs reduce the biofilm formation by two to fourfold compared to free SeNPs. However, free niosome as a control did not have anti-biofilm effects. One of the reasons that niosomes have more anti-biofilm effects is their significant antimicrobial effects in such a way that with the increase in the percentage of bacterial death, the number of bacteria does not reach the level that can form a biofilm, and as mentioned, the increase in antimicrobial effects is due to the fusion of niosomes with the bacterial cell membrane^47^. Kashef et al. loaded ciprofloxacin into niosomes and studied its anti-biofilm effects against biofilm-forming strains of *S. aureus*. The results of this study showed that niosome-loaded ciprofloxacin can reduce MIC value by 2–8 times and can significantly reduce biofilm formation at a concentration of 1/8 MIC^48^. Abu-Elghait et al. synthesized tertiary composite based on cellulose and myco-synthesized selenium nanoparticles and investigated its anti-biofilm effects against *Pseudomonas aeruginosa* and *Staphylococcus aureus* strains. The results showed that this myco-synthesized novel cellulose-based selenium nanoparticles tertiary composite can inhibit biofilm and have anti-biofilm effects^49^.

### Biofilm gene expression analysis

**Figure 6:**
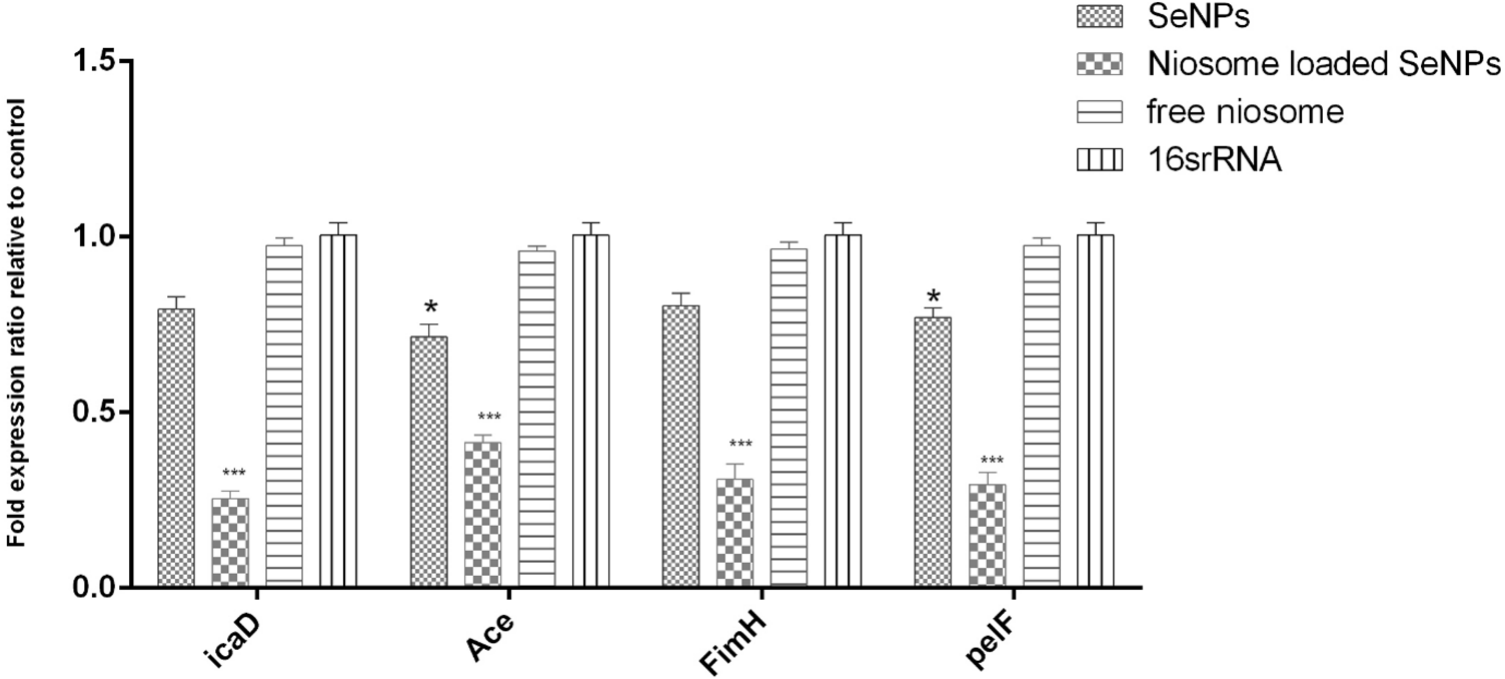
Biofilm-related gene expression analysis after treatment with free SeNPs, free niosome, and niosome loaded SeNPs. n = 3, ***p < 0.0001; **p < 0.01; *p < 0.0.5.

To study the effects of niosomes-loaded SeNPs, free SeNPs, and free niosome on the expression level of biofilm-forming genes, the Real-Time PCR method was used. First, the strains were treated with their sub-MIC concentrations and after RNA extraction and cDNA synthesis, the expression levels of *icaD*, *Ace*, *fimH*, and *pelF* biofilm genes were measured. The results showed that the expression level of biofilm-forming genes in the strains treated with niosome-loaded SeNPs was significantly reduced compared to SeNPs and free niosome (Fig. 6). One of the reasons that probably niosomes-loaded SeNPs can reduce the expression of biofilm genes is the binding of SeNPs to transcription factors and inhibiting the expression of biofilm genes^50^.

### In vitro cytotoxicity

**Figure 7:**
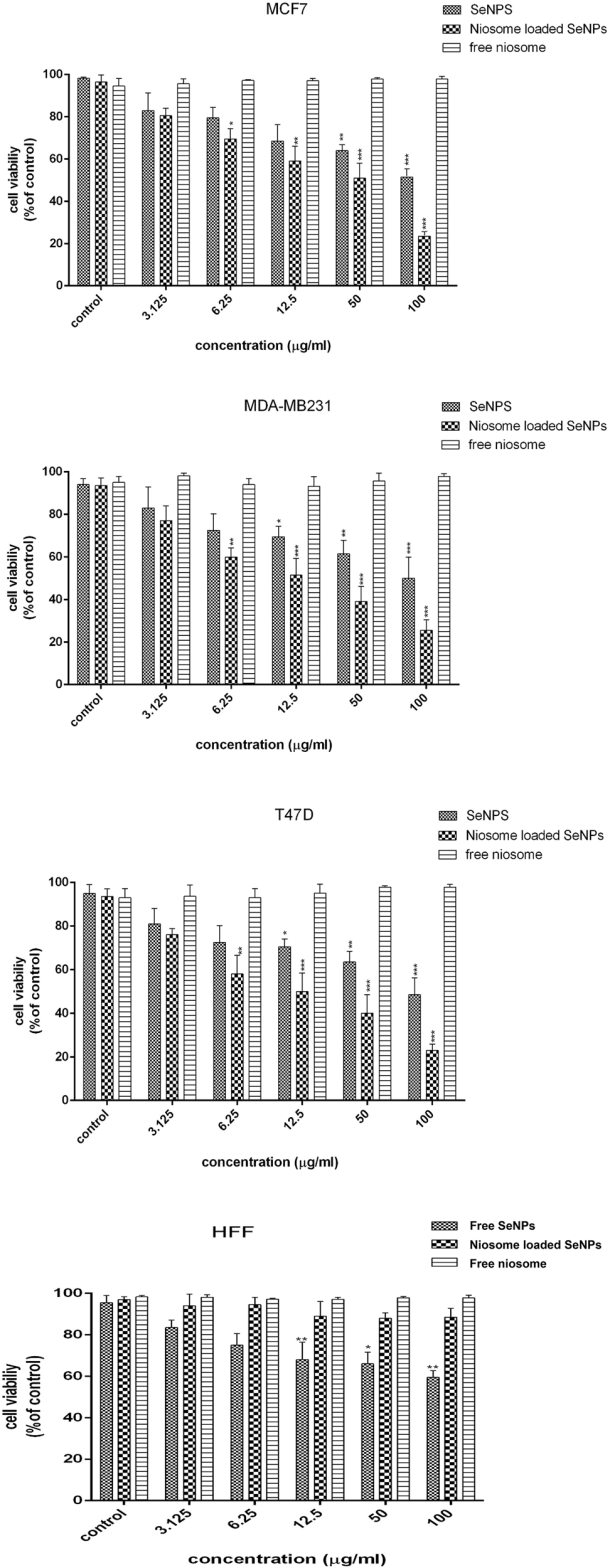
In vitro cytotoxicity of free SeNPs, niosome loaded SeNPs, and free niosome on MCF-7, T47D MDAMB231, and normal HFF cell. n = 3, *** *p* < 0.0001; **p < 0.01; *p < 0.0.5.

The cytotoxicity results of free SeNPs, free niosome, and niosome-loaded SeNPs on breast cancer cell lines in 24 h showed that niosome-loaded SeNPs had the greatest cytotoxic effect in comparison to free SeNPs (Fig. 7). The results showed that at the highest concentration (100 μg/mL), the cell survival rate in MCF-7, T47D, and MDAMB231 cells treated with niosomes loaded SeNPs was 23.65 ± 1.21, 31.25 ± 1.13%, and 23.35 ± 1.36%, respectively, which was much lower than free SeNPs, which shows the significant cytotoxic effects of niosomes-loaded SeNPs. Also, the niosome-loaded SeNPs had dose-dependent cytotoxicity, and the cell survival rate decreased with the increase in the dose. Compared to niosomes-loaded SeNPs, free SeNPs had more cytotoxic effects against the HFF normal cell line, one of the reasons for which could be the presence of some herbal compounds on SeNPs. Also, the cytotoxicity results of free niosome against breast cancer and HFF normal cell lines showed that it has no significant cytotoxic effects on cell lines and therefore it is biocompatible. Various studies have been conducted on investigating the anti-cancer effects of niosomes containing nanoparticles and antimicrobial drugs. Rezaie Amale et al. loaded gold nanoparticles into niosomes and evaluated their cytotoxic effects against a human ovarian cancer cell line. The results of this study indicated that niosomes-loaded gold nanoparticles had dose-dependent cytotoxicity effects and had more significant cytotoxic effects than free gold nanoparticles^51^. Xuan et al. synthesized a co-delivery system consisting of SeNPs@liposome and investigated its cytotoxic effects against lung cancer (A549) and cervical cancer (HeLa) cell lines using the MTT assay. The results of this study demonstrated that SeNPs@liposome can increase the effects of cytotoxicity against cancer cell lines. All the results of other researchers are consistent with the results of our study in that the cytotoxic effects of SeNPs increase when they are loaded into a drug delivery system^52^.

### Apoptosis gene expression analysis

**Figure 8:**
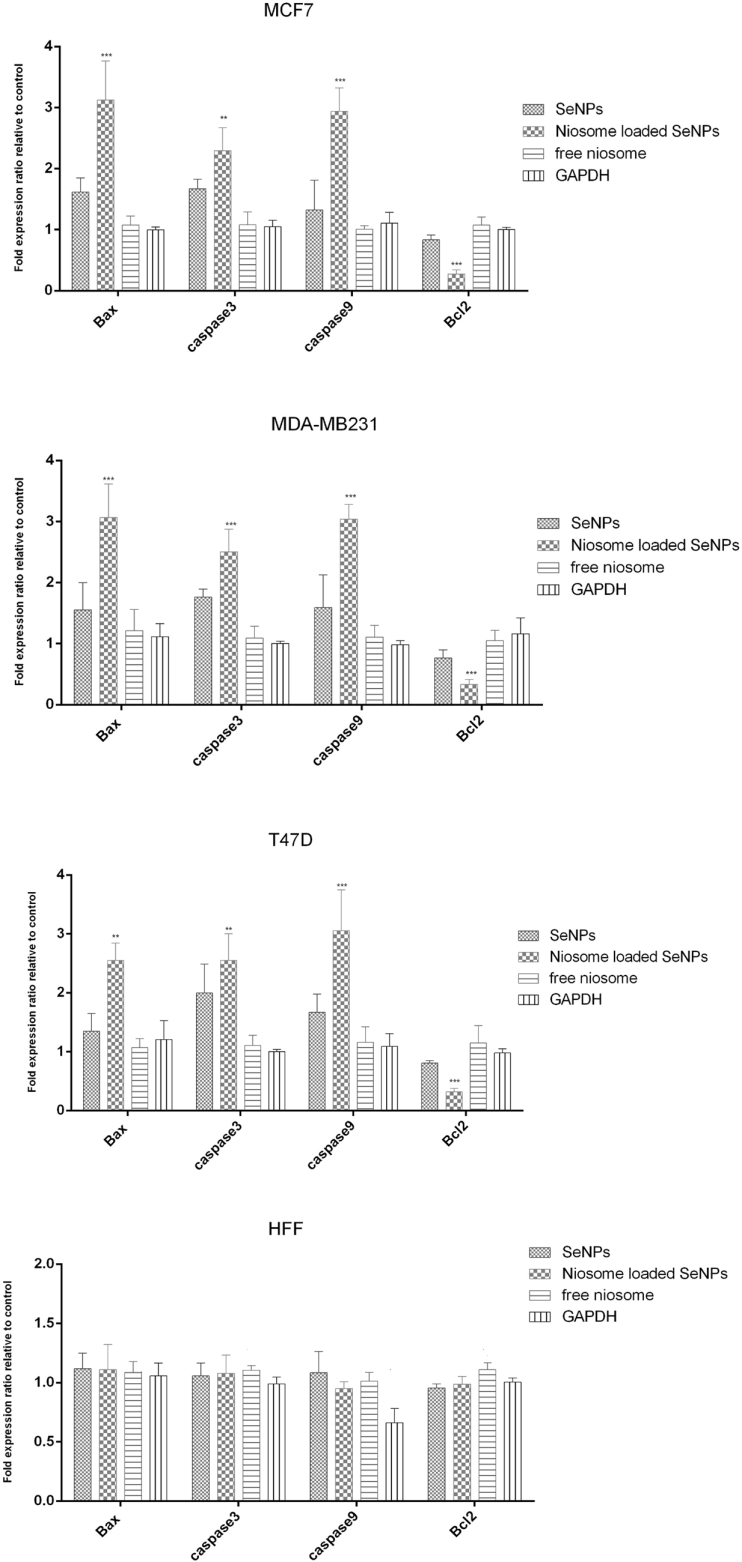
Apoptosis gene expression analysis in breast cancer cell lines and normal cells after treatment with free niosome, free SeNPs, and noisome-loaded SeNPs. n = 3, ***p < 0.0001; **p < 0.01.

The expression of apoptotic genes including *Bax*, *cas3*, *casp9*, and *Bcl2* in MCF-7, T47D, and MDAMB231 cells treated with the IC50 concentration of niosomes loaded SeNPs and free SeNPs was evaluated using the Real-Time PCR method. The Real-Time PCR data analysis was performed based on the cycle threshold (Ct) comparison. In this study, the difference in the Ct obtained from the tested samples (cells treated) and the control samples (not treated cells) was calculated and the gene expression was calculated using the ΔΔCt formula (the ratio of the target gene to the reference gene (beta-actin) was calculated through 2^−ΔΔCt^). The results showed that the expression of *Bax*, *cas3*, and *cas9* genes compared to the reference gene in breast cancer cell lines was up-regulated significantly within 24 h compared to free SeNPs. Also, the level of *Bcl2* gene expression in breast cell lines treated with niosomes-loaded SeNPs down-regulated significantly compared to free SeNPs (Fig. 8). Gharbavi et al. prepared a hybrid system of SeNPs@niosome and studied its cytotoxic and apoptotic effects against a lung cancer cell line (A549). The results of this study demonstrated that niosomes loaded SeNPs enhances cytotoxicity and upregulate the *Bax* apoptotic gene expression and down-regulate the *Bcl2* gene^53^. Akbarzadeh et al., synthesized curcumin-loaded niosome@calcium alginate nanocarrier and investigated its cytotoxic effects against breast cancer cell lines including MDA-MB-231 and SKBR3, and analyzed the expression level of apoptotic genes such as *P53*, *Bax*, *caspase-3*, and *caspase-9*. The results of this study revealed that niosomes have significant cytotoxic effects on breast cancer cell lines and the expression of apoptotic genes in the studied cell lines upregulated significantly^54^. Dabbagh Moghaddam et al. prepared niosomes loaded melittin and studied its apoptotic effects against breast cancer cell lines such as 4T1 and SKBR3. The results showed that niosomes loaded melittin can increase the expression of apoptotic genes including *Bax*, caspase3, and *caspase9*, and decrease the expression of *Bcl2*, *MMP2*, and *MMP9* genes, which indicated the induction of apoptosis by niosomes loaded melittin^55^.

## Conclusion

The results of this study demonstrate the effectiveness of successfully biosynthesized SeNPs that *T. cherleri* extract act as a capping and stabilizing agent. In this study, for the first time, SeNPs were loaded into noisome and were characterized using UV–vis spectroscopy, FTIR, XRD, SEM, EDX, and TEM analysis. Moreover, their antimicrobial, anti-biofilm, and anti-cancer effects against breast cancer cell lines were investigated. The noisome-loaded SeNPs showed significant antibacterial and anti-biofilm effects against pathogenic bacteria and cytotoxic effects against breast cancer cell lines. Also, niosomes loaded SeNPs can down-regulated the expression of biofilm-related genes in pathogenic bacteria and up-regulated the expression of apoptotic genes in breast cancer cell lines. Therefore, it can induce apoptosis in breast cancer cell lines, which is a desirable feature for nanomedicines. Overall, it can be concluded that niosomes can be used as a suitable drug delivery system for therapeutic purposes.

## Data Availability

The datasets used and/or analyzed during the current study are available from the corresponding author on reasonable request and can be available from the corresponding author on request.
